# 巨大原发性肺软骨瘤1例

**DOI:** 10.3779/j.issn.1009-3419.2011.04.15

**Published:** 2011-04-20

**Authors:** 小明 邱, 大兴 朱, 军 陈, 清华 周

**Affiliations:** 300052 天津，天津医科大学总医院肺部肿瘤外科，天津市肺癌研究所 Department of Lung Cancer Surgery, Tianjin Lung Cancer Institute, Tianjin Medical University General Hospital, Tianjin 300052, China

## 1 临床资料

患者，女，40岁，查体发现右下肺肿物1周入院。既往体健。体格检查：体温正常，脉搏、血压、呼吸均无异常。胸壁无压痛，双肺呼吸运动对称，触觉语颤对称，叩诊呈清音，听诊双肺呼吸音稍粗，未闻及干、湿啰音。入院后CT检查：右下肺后基底段可见大小约6.4 cm×5.7 cm×7.5 cm的不规则软组织肿块，内可见多发斑片钙化，形态不规则，肿块与局部胸膜粘连，未见明显强化（图 1）。完善术前检查后于2010年8月17日在全麻下行剖胸探查，术中见包块位于右肺下叶后段，突出于肺表面，不规则分叶，被覆脏层胸膜及少量肺组织，与周围组织无粘连，质硬。行右肺下叶楔形切除术，完整切除肿物。包块为灰白灰褐不规则组织，大小6.5 cm× 6.0 cm×5.0 cm，有多个结节状突起，切面质地较硬，灰白色，半透明状，软骨样（图 2）。镜检：瘤体由较成熟的软骨细胞构成，周围为软骨基质包绕，呈不规则分叶状（图 3）。病理诊断：右下肺软骨瘤，局部生长活跃。患者术后10天治愈出院。

**1:**
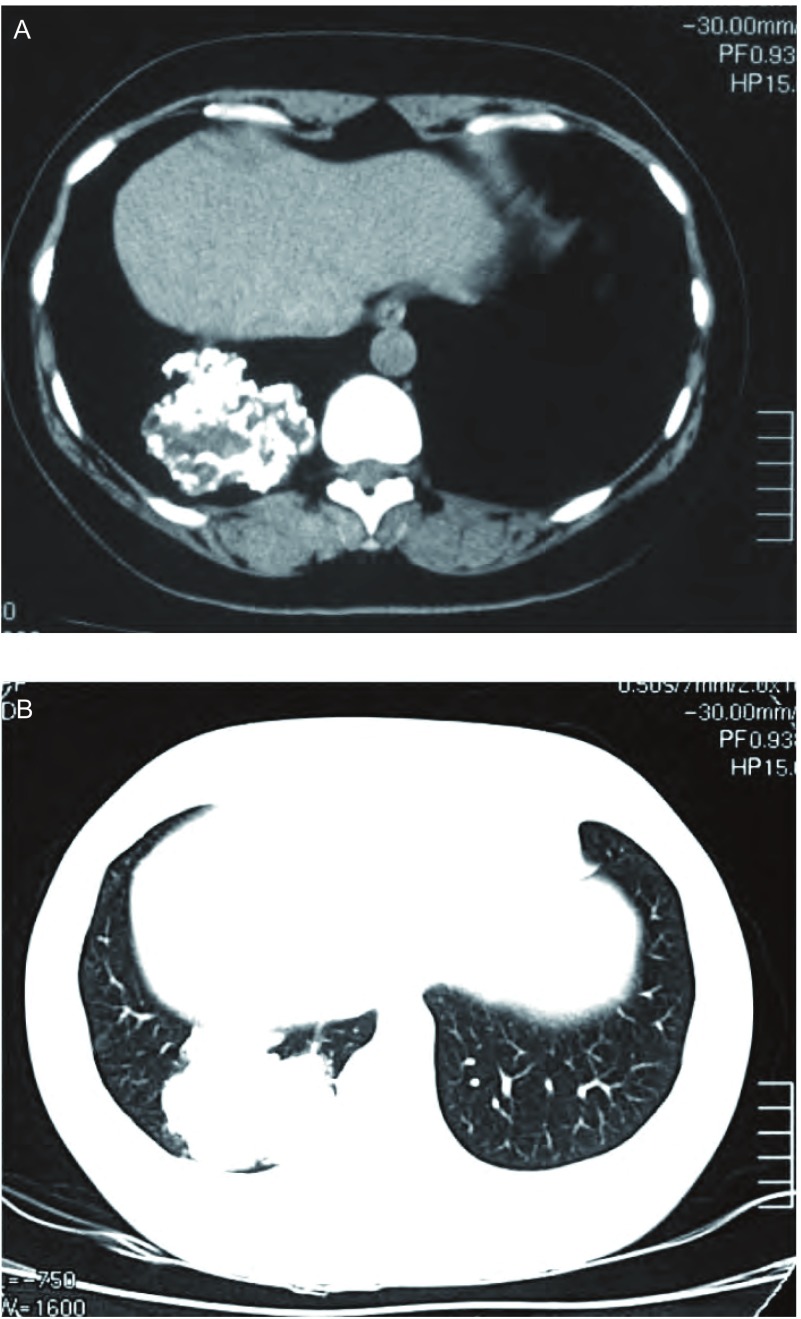
胸部CT检查。A：纵隔窗显示右下肺软组织肿物，内见多发钙化；B：肺窗显示肿物位于右下肺内。 CT scan of the chest. A: in mediastinal window, the tumor was located in the right lower lobe, multiple calcifications were existed in the tumor; B: in lung window, the tumor is in the parenchyma of right lower lobe.

**2:**
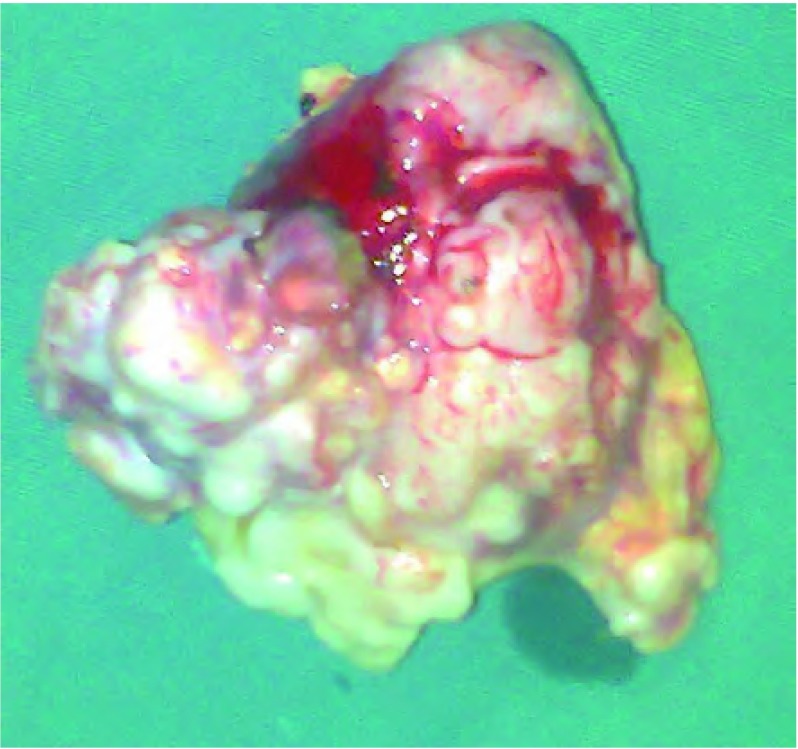
大体标本，包块为灰白灰褐不规则组织 Grossly, tumor was irregular shaped, with grayish brown color

**3:**
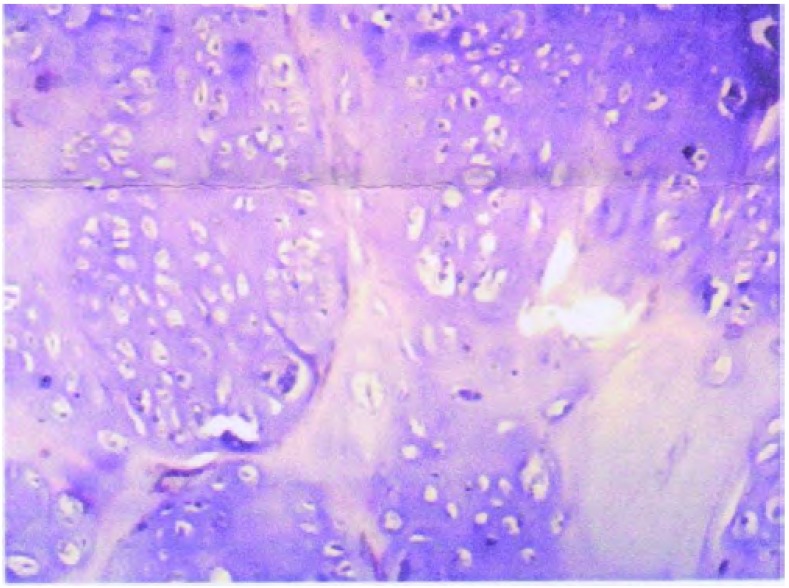
瘤体由较成熟的软骨细胞构成，周围为软骨基质包绕（HE，×40） The tumor was constituted of relatively mature chondrocytes which are surrounded by cartilage matrix (HE, ×40)

## 2 讨论

软骨瘤是位于支气管软骨部最常见的支气管肿瘤，但位于肺实质内者少见。该病多合并Carney综合征^[1]^，即3种不同的脏器同时发生3种不同的肿瘤：胃平滑肌肉瘤、肾上腺外嗜铬细胞瘤和肺软骨瘤。临床上大部分患者只表现为2种或1种肿瘤^[2]^，该病例术前头部MRI，上腹部强化CT均未见异常。临床上有患者最长26年后发现第2个肿瘤，这类患者仍建议术后密切随访^[2]^。

原发性肺软骨瘤迄今病因未明，一般认为肺软骨瘤可能来源于胚胎时期气管软骨的发育异常，肺中心发育成软骨的间叶组织分散到肺实质，随着肺的发育逐步形成软骨瘤^[3]^。因此肺软骨瘤多位于两肺周边的肺实质内，本例患者即位于右下肺胸膜下。

肺软骨瘤好发于女性（85%），发病年龄为7岁-59岁，多数患者（82%）在30岁前确诊^[2]^，本例患者也在此年龄范围，但是由于肿瘤位于肺周边，患者一直无胸闷、胸痛、咳嗽等症状，查体做胸片检查才发现，病变可能已存在多年，并生长到较大体积。肺软骨瘤全部由软骨细胞构成，可为透明软骨、纤维软骨或弹力软骨，或由各种软骨混合构成，其间可有纤维分隔，有明显的软骨陷窝。软骨组织通常部分钙化或者骨化现象明显。胸片及CT多表现为肺野外带圆形或椭圆形密度均匀的孤立结节或肿块，肿瘤内可见斑点状钙化；与周围组织界限清，增强扫描时有轻度强化。本例患者标本完全符合上述表现，而且由于包块巨大，生长时间较长，钙化斑片明显。组织中无脂肪组织，平滑肌和呼吸上皮组织嵌入其中，这可与发生在大支气管的软骨样错构瘤相鉴别^[4]^。

由于其症状不明显以及无特异性影像学改变，软骨瘤的术前诊断较为困难，确诊依靠手术病理检查。由于存在恶变的可能^[5]^，手术完整切除为肺软骨瘤首选的治疗方法，临床上多采用肺段切除、肺叶切除或肺楔形切除。
